# Condylar Bone Quality in Growing Children Is Associated With Genetic Polymorphisms in Genes Involved in Calcium and Phosphate Maintenance

**DOI:** 10.1155/bmri/9337029

**Published:** 2026-01-07

**Authors:** Erika Calvano Küchler, Caio Luiz Bitencourt Reis, Gabriela Fonseca-Souza, Daniel Hemming, Flares Baratto-Filho, Cristiano Miranda de Araujo, Svenja Beisel-Memmert, Juliana Feltrin-Souza, Michelle Nascimento Meger, Bianca Lopes Cavalcante-Leão

**Affiliations:** ^1^ Department of Orthodontics, Medical Faculty, University Hospital Bonn, Bonn, Germany, uni-bonn.de; ^2^ School of Dentistry, Federal University of Alfenas, Alfenas, Minas Gerais, Brazil, unifal-mg.edu.br; ^3^ Department of Stomatology, Federal Paraná University, Curitiba, Paraná, Brazil; ^4^ School of Dentistry, Tuiuti University of Paraná, Curitiba, Paraná, Brazil; ^5^ School of Dentistry, Univille University, Joinville, Santa Catarina, Brazil, univille.edu.br

**Keywords:** fractal dimension analysis, mandibular condyle, panoramic radiography, vitamin D receptor

## Abstract

Single nucleotide polymorphisms (SNPs) play a crucial role in regulating vitamin D, parathyroid hormone (PTH), and calcitonin concentrations, which are involved in bone health. Some reports suggested that fractal analysis is useful in the morphometric analysis of the mandible trabecular bone in panoramic radiographs. Therefore, we investigated if SNPs in genes that influence vitamin D, calcitonin, and PTH levels are involved in condylar bone quality during the active growing phase of the mandible. Fractal dimension was obtained from the condyle region of interest (ROI) using panoramic radiographs and used to measure the complexity and the microarchitecture of the bone. Fractal dimension using the box‐counting algorithm was then calculated. In order to avoid information bias, a script to automate the commands in the software ImageJ was generated to ensure consistency and minimize the potential for human error during the data analysis process. SNPs in vitamin D receptor (*VDR*), cytochrome P450 family 27 subfamily B member 1 (*CYP27B1*), cytochrome P450 family 24 subfamily A member 1 (*CYP24A1*), vitamin D binding protein (*VDBP*), SEC23 homolog A (*SEC23A*), calcitonin receptor (*CALCR*), and parathyroid hormone (*PTH*) were analyzed. DNA extracted from saliva was used for genotyping analysis of *VDR* (rs7975232, rs2228570, and rs1544410), *CYP27B1* (rs4646536), *CYP24A1* (rs927650), *VDBP* (rs4588), *SEC23A* (rs8018720), *CALCR* (rs1801197), and *PTH* (rs6256, rs307247, and rs694). A statistical analysis was performed with an alpha error tolerance of 5%. A total of 100 children were included; 50 (50%) were boys and the age ranged from 5 to 14 years old. Fractal dimensions were compared among genotypes. The GT (mean = 1.20 and standard error = 0.03, *p* = 0.024) and TT genotypes (mean = 1.16 and standard error = 0.06, *p* = 0.047) in the gene VDBP (rs4588) presented lower fractal dimension. The GG genotype in SEC23A (rs8018720) (mean = 1.34 and standard error = 0.03, *p* = 0.011) and the TC genotype in PTH (rs694) showed an increased fractal dimension (mean = 1.29 and standard error = 0.03, *p* = 0.020). In conclusion, SNPs in VDBP, SEC23A, and PTH encoding genes are associated with mandibular condylar trabecular bone structure in children.

## 1. Introduction

The condyle is the key anatomic structure of the mandible that connects with the temporal bone of the skull and forms the temporomandibular joint (TMJ). The mandibular condyle is the hinge point for the mouth to open, swallow, breathe, phonate, suck, and perform different facial expressions. The condyle is formed by trabecular and cortical bone, and its complex movements, in different orthogonal planes and multiple axes of rotation, work in synergy with all the TMJ structures [1]. The condyle is a highly adaptive structure, mainly during the active growing phase of the mandible, which occurs until about 25 years old [2, 3]. Local and intrinsic factors, such as sex, hormones, and genetics, may influence the bone trabeculae quality and potentially increase the risk of TMJ dysfunctions, such as condyle resorption and hyperplasia [4].

Three critical hormones are substantially studied as crucial factors in the bone maintenance molecular pathway: vitamin D, parathyroid hormone (PTH), and calcitonin. These hormones work together essentially to regulate circulating calcium and phosphate levels. Vitamin D (specifically its active form, calcitriol) increases calcium and phosphate absorption in the intestines, which is crucial for bone mineralization. It stimulates osteoblasts and aids in the maintenance of proper bone density [5]. PTH promotes the conversion of vitamin D into its active form (calcitriol) in the kidneys, improving calcium absorption. PTH is released to increase calcium availability and activates osteoclasts to release calcium [6]. Calcitonin is released by the thyroid gland when calcium levels are high and it suppresses osteoclast activity, reducing calcium release from bones [7].

Serum vitamin D, PTH, and calcitonin concentrations are strongly influenced by genetics [8]. The vitamin D receptor (*VDR*) gene, which encodes the vitamin D nuclear receptor and acts as a transcription nuclear factor, plays a key role in regulating calcium and phosphate homeostasis and bone metabolism. It is activated by calcitriol (the active form of vitamin D) and influences the expression of genes involved in calcium and phosphate absorption in the intestines and bone metabolism by regulating osteoblast and osteoclast activity [9]. Genes such as cytochrome P450 family 27 subfamily B member 1 (*CYP27B1*), cytochrome P450 family 24 subfamily A member 1 (*CYP24A1*), vitamin D binding protein (*VDBP*), SEC23 homolog A (*SEC23A*), calcitonin receptor (*CALCR*), and *PTH* are also important genes involved in vitamin D, calcitonin, and PTH levels [10–19].

Recent evidence indicates that single nucleotide polymorphisms (SNPs) play a crucial role in regulating vitamin D, PTH, and calcitonin concentrations [10–12, 16–20]. In this way, we hypothesized that SNPs associated with the concentrations of these hormones may be associated with the condyle bone quality. To evaluate condyle bone quality, it is possible to apply methods to measure the microarchitecture of trabecular bone. Fractal analysis is a method employed to quantify bone complexity through a metric that is known as fractal dimension (FD), and it is a method reported in the literature for the quantitative morphometric bone evaluation [21]. Some reports suggested that fractal analysis could be useful in the morphometric analysis of the mandible trabecular bone in panoramic radiographs [22–26]. Therefore, we investigated if SNPs in genes that influence vitamin D, calcitonin, and PTH levels are involved in condylar bone quality during the active growing phase of the mandible.

## 2. Materials and Methods

This phenotypic–genotypic cross‐sectional study was designed and reported following the STREGA reporting guidelines [27]. The project followed the Declaration of Helsinki and was previously approved by the Committee for Ethics in Research in Human Beings of the Health Sciences of the Federal University of Parana (Protocol Number 3.752.172).

The recruitment was performed in a dental school, in which children and their legal guardians were invited and clarified about the details of the project. The legal guardians who accepted that the minor take part in the project consented by providing a handwritten signature. Children received a document suitable for their age and signed it with a handwritten signature. All included patients and legal guardians were capable of reading and writing.

To minimize differences in sun exposure, the study included only children from the Curitiba region of Brazil. Children who presented underlying syndromes, facial trauma, facial anomalies, severe systemic disorders, and oral cleft were excluded.

### 2.1. Panoramic Analysis

FD was obtained from the condyle region of interest (ROI) using panoramic radiographs. FD was used to measure the complexity and irregularity of complex structures like the microarchitecture of bone. FD was calculated to generate a single numerical value that summarizes bone structure complexity, in which values that are close to 0 suggest a loss of bone complexity, demonstrating less interconnected trabeculae, whereas values near 2 show a more intricate and well‐connected trabecular structure reflecting healthier bone architecture.

**Figure 1:**
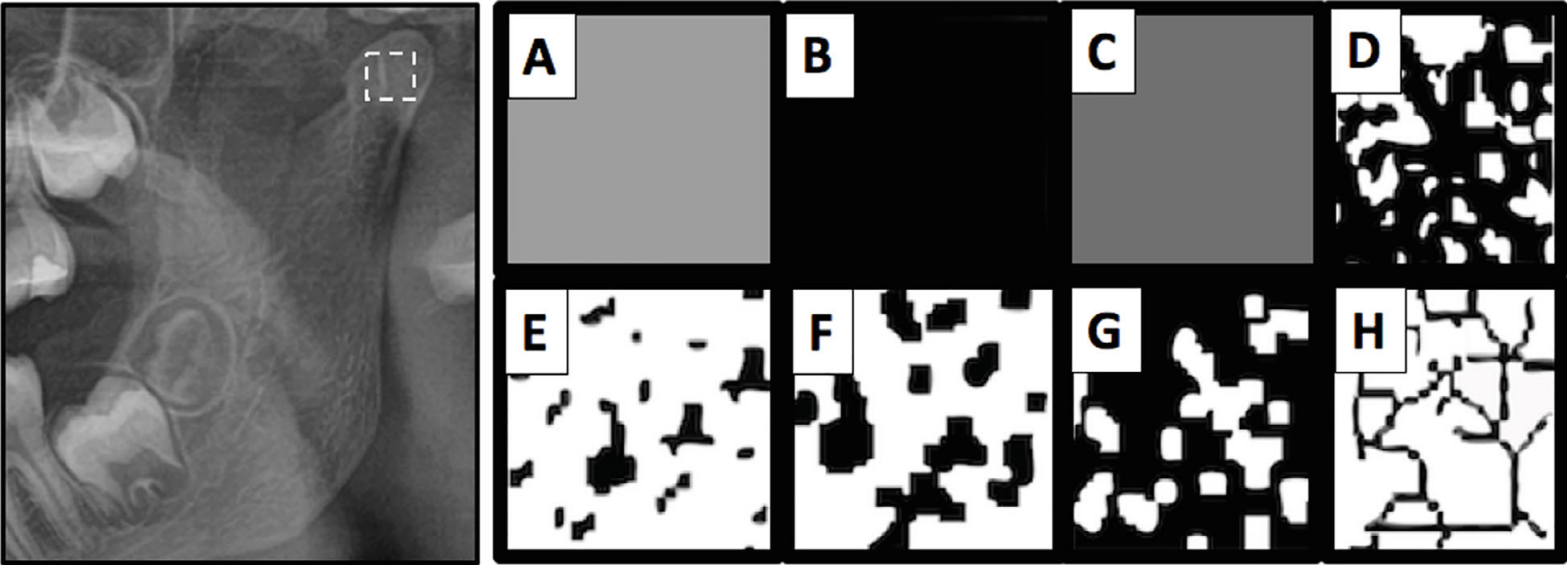
Region of interest on condyle and fractal analysis process: A cropped region of interest and stages of FD analysis.

All panoramic radiographs were high quality and were examined digitally. The examination was conducted in a darkroom by a single previously trained examiner. The TIF (Tagged Image File) of panoramic radiographs was standardized in width and height of 2976 × 1536 pixels. The ROI was selected from both sides (right and left) of the condyles as shown in Figure 1. A square form with 50 × 50 pixels placed in the geometric center of the head of the condyle was used to determine the ROI.

FD was assessed using the software ImageJ Version 1.53 (National Institutes of Health). The images underwent a series of steps, which started with Gaussian blur applied in two dimensions using a sigma of 2.0. The images were binarized focusing on pixels with grey values ranging from 177 to 255 that were subsequently subjected to skeletonization. FD using the box‐counting algorithm was calculated using the plugin BoneJ Version 1.4.3 [28].

Interexaminer and intraexaminer tests were performed. The examiner was trained and calibrated by a senior researcher. Both the examiner and the senior researcher accessed the ROIs from panoramic radiographs of 15 patients. These patients were not included in the sample of the study. The reliability measured using the intraclass correlation coefficient (ICC) was calculated after a 2‐week interval. The result indicated a strong interexaminer reliability of ICC = 0.85. The examiner re‐evaluated the same 15 panoramic radiographs to assess intraexaminer reliability, and the result also indicated a strong interexaminer reliability (ICC = 0.83). The examiner assessed a maximum of 15 panoramic radiographs per day over a period of at least 60 min.

In order to avoid information bias, a script to automate the commands in the software ImageJ was generated to ensure consistency and minimize the potential for human error during the data analysis process.

### 2.2. SNP Selection, DNA Isolation and Genotyping

For the current study, SNPs were screened from the dbSNP database (http://www.ncbi.nlm.nih.gov/snp/) and SNPinfo (http://snpinfo.niehs.nih.gov/). The characteristics of the SNPs are shown in Table 1.

**Table 1:** Characteristics of the studied SNPs.

**Acronym**	**Gene name**	**SNP**	**Base change**	**Role of the SNP and reference**	**GSR**	**x** ^**2HWE**^
*VDR*	*Vitamin D receptor*	rs7975232	A/C	Increase of plasmatic levels of vitamin D [10]	0.96	2.68
		rs2228570	G/A	Decrease of plasmatic levels of vitamin D [11]	0.97	0.51
		rs1544410	C/T	Increase of plasmatic levels of vitamin [12]	0.97	0.16
*CYP27B1*	*Cytochrome P450 family 27 subfamily B member 1*	rs4646536	A/G	Increase of plasmatic levels of vitamin [13]	0.92	0.01
*CYP24A1*	*Cytochrome P450 family 24 subfamily A member 1*	rs927650	C/T	Increase of plasmatic levels of vitamin [14]	0.96	0.84
*VDBP*	*Vitamin D binding protein*	rs4588	G/T	Decrease of plasmatic levels of vitamin D [15]	0.97	0.33
*SEC23A*	*SEC23 homolog A*	rs8018720	C/G	Decrease of plasmatic levels of vitamin D [16]	0.96	0.01
*CALCR*	Calcitonin receptor	rs1801197	A/G	Involved in bone mineral quality [20]	0.96	2.87
*PTH*	*Parathyroid hormone*	rs6256^a^	G/T	Decrease of PTH serum levels [18]	0.96	2.07
		rs307247	A/G	Decrease of PTH serum levels [19]	0.97	0.48
		rs694	C/T	Decrease of PTH serum levels [17]	0.97	0.42

Abbreviations: GSR, genotyping success rate; HWE, Hardy–Weinberg equilibrium; SNP, single nucleotide polymorphism.

^a^Stop gained.

A 5‐mL saline solution 0.9% mouthwash was used to collect the saliva for the genomic DNA isolation from epithelial cells following a published protocol [29]. The DNA quality and concentration of each sample were assessed using a NanoDrop 2000 (Thermo Scientific, Wilmington, Delaware, United States). The allelic discrimination analysis (genotyping) was made through real‐time polymerase chain reaction in the StepOnePlus Real‐Time PCR System (Thermo Fisher Scientific, Waltham, Massachusetts, United States). The reaction comprised 3.1 *μ*L total volume (1.5 *μ*L of DNA—5 ng/*μ*L), 1.5 *μ*L of TaqMan Genotyping Master Mix, and 0.095 *μ*L of TaqMan probes (Applied Biosystems, Foster City, California, United States). A negative control template was included in each experimental plate, and 10% of the samples were randomly selected for repeated analysis in different plates. The results showed 100% concordance of the genotypes randomly repeated in different reactions. DNA samples that failed to amplify were excluded from statistical analyses. The genotyping success rate is shown in Table 1 per SNP.

### 2.3. Sample Size Calculation

The sample size calculation was performed based on the mean difference between two independent means of 0.2 (standard deviation of 0.25), using the parameters of alpha = 5*%* and power = 80*%*. The calculation predicts a minimum of 16 patients per genotype. Taking into consideration that some SNPs have low frequency, the total sample was estimated at 100 patients.

### 2.4. Statistical Analysis

Pearson′s chi‐square (*χ* ^2^) test without correction was used to calculate the Hardy–Weinberg equilibrium (HWE).

Two ROIs (right and left condyles) were obtained from each participant (multiple observations per participant). In this way, we adjusted the statistical analysis through “Complex samples” module in IBM SPSS for Windows Version 25.0 (IBM, Chicago, Illinois, United States). This module applies appropriate statistical techniques to accommodate multiple observations per participant. First, the module identifies the elements of complex sampling, which are the left and right condyle per individual. Second, the module performs the one‐factor analysis of variance (ANOVA) considering the elements separately, with equal probability and PPS (probability proportionate to size) adjustment methods. Last, a generalized linear model (GLM), assuming a normal distribution with a log link function, was performed to evaluate the FD differences between genotypes. The analysis was adjusted by sex and age, as these variables could be confounding factors. The alpha error tolerance was 5%. Mean and standard error (SE) were calculated for each genotype.

## 3. Results

A total of 100 children were included; 50 (50%) were boys and 50 (50%) were girls. The mean age of the included children was 8.5 (standard deviation = 2), and the age ranged from 5 to 14 years old.

Chi‐square (*χ* ^2^) analysis showed that all the evaluated SNPs were in HWE, in which all *χ* ^2^ ≤ 3.841. More details of the HWE per SNP are shown in Table 1.

FD was compared among genotypes for all SNPs and the results are presented in Table 2. The heterozygous GT genotype (mean = 1.20 and SE = 0.03, *p* = 0.024) and homozygous TT genotype (mean = 1.16 and SE = 0.06, *p* = 0.047) in the gene *VDBP* (rs4588) were both statistically significantly associated with a decrease in FD compared to the homozygous GG genotype (mean = 1.28 and SE = 0.02). The homozygous GG genotype in SEC23A (rs8018720) was statistically significantly associated with increased FD (mean = 1.34 and SE = 0.03, *p* = 0.011) compared to the homozygous genotype CC (mean = 1.24 and SE = 0.02). The FD was also statistically significantly associated with the heterozygous TC genotype in the gene *PTH* (rs694), which showed increased FD (mean = 1.29 and SE = 0.03, *p* = 0.020) compared to the homozygous genotype TT (mean = 1.20 and SE = 0.03).

**Table 2:** Fractal dimensions according to the genotypes.

**SNP**	**N**	**%**	**Mean**	**Std. error**	**95% CI**		**p** **value**
					**Lower**	**Upper**	
*VDR* rs7975232							
AA	36	37.5	1.25	0.03	1.20	1.30	Reference
AC	39	40.6	1.22	0.03	1.17	1.27	0.430
CC	21	21.9	1.27	0.04	1.20	1.34	0.638
*VDR* rs2228570							
GG	38	39.2	1.24	0.03	1.19	1.30	Reference
AG	48	49.5	1.24	0.02	1.19	1.29	0.969
AA	11	11.3	1.25	0.04	1.18	1.33	0.757
*VDR* rs1544410							
CC	40	41.3	1.26	0.02	1.21	1.31	Reference
TC	46	47.4	1.22	0.02	1.17	1.27	0.255
TT	11	11.3	1.26	0.05	1.16	1.37	0.934
*CYP27B1* rs4646536							
AA	50	54.4	1.22	0.02	1.18	1.27	Reference
AG	36	39.1	1.27	0.03	1.22	1.33	0.172
GG	6	6.5	1.18	0.10	0.99	1.37	0.651
*CYP24A1* rs927650							
CC	21	21.7	1.25	0.04	1.18	1.33	Reference
CT	53	54.6	1.23	0.02	1.19	1.28	0.632
TT	23	23.7	1.25	0.04	1.18	1.32	0.998
*VDBP* rs4588							
GG	56	57.7	1.28	0.02	1.24	1.32	Reference
GT	34	35.1	1.20	0.03	1.14	1.25	0.024 ^∗^
TT	7	7.2	1.16	0.06	1.05	1.27	0.047 ^∗^
*SEC23A* rs8018720							
CC	66	68.8	1.24	0.02	1.20	1.28	Reference
CG	27	28.1	1.24	0.03	1.18	1.31	0.854
GG	3	3.1	1.34	0.03	1.28	1.41	0.011 ^∗^
*CALCR* rs1801197							
AA	42	43.8	1.24	0.02	1.19	1.29	Reference
AG	37	38.5	1.26	0.03	1.20	1.31	0.760
GG	17	17.7	1.20	0.03	1.14	1.27	0.328
*PTH* rs6256							
GG	71	73.9	1.23	0.02	1.20	1.27	Reference
GT	21	21.9	1.28	0.03	1.22	1.34	0.188
TT	4	4.2	1.16	0.10	0.96	1.37	0.513
*PTH* rs307247							
GG	34	35.0	1.27	0.03	1.21	1.32	Reference
GA	44	45.4	1.23	0.02	1.18	1.27	0.288
AA	19	19.6	1.24	0.04	1.15	1.32	0.583
*PTH* rs694							
TT	30	30.9	1.20	0.03	1.15	1.26	Reference
TC	45	46.4	1.29	0.03	1.24	1.34	0.020 ^∗^
CC	22	22.7	1.20	0.03	1.14	1.27	0.945

*Note:* Adjusted by age and gender.

Abbreviation: CI, confidence interval.

^∗^denotes statistical significance.

## 4. Discussion

In the current study, we hypothesized that vitamin D, calcitonin, and PTH‐related genes are involved in the human mandibular condylar bone quality. Several studies investigated the properties of the mandibular condylar bone in different conditions; however, to the best of our knowledge, this is the first study to investigate the genetic factors involved in the morphometric patterns of the mandibular condylar bone in humans. We used fractal analysis, which is a method for the quantitative morphometric evaluation of bone, to calculate the quality and structural integrity of bone in the condyle region in children′s panoramic radiographs. We observed that SNPs in *VDBP* (rs4588), *SEC23A* (rs8018720), and *PTH* (rs694) were associated with FD in the condyle. The understanding of the role of vitamin D, calcitonin, and PTH‐related genes in mandibular condylar bone is important once bone tissue characteristics in this anatomical region play a critical role in determining the mechanical stability of the TMJ under the macrolevel loading [30].

Although panoramic radiographic has some limitations, these image exams have been used to assess FD in several previous studies [22–26]. A systematic review from 2025 [31] was performed to screen the application and effectiveness of fractal analysis in evaluating TMJ with dental image exams. The authors included 15 primary studies and concluded that FD using dental images, including panoramic radiographs, is a valuable tool for detecting early degenerative changes in the condyle region. More recently, fractal analysis was used to evaluate the effect of phenylketonuria on mandibular bone in panoramic radiographs [22]. These show the applicability of panoramic radiographs for fractal analysis of the condyle region.

The biological actions of vitamin D occur by the binding of its nuclear receptor, the VDR [32], which is expressed in the parathyroid glands and acts as sensors for vitamin D level detection and maintenance, adjusting PTH release and synthesis [33]. Furthermore, other molecules such as the enzymes CYP24A1, CYP27B1, VDBP, and SEC23A are pivotal determinants of the local levels of vitamin D [34]. VDBP is a multifunctional glycoprotein primarily involved in transporting vitamin D and its metabolites in the bloodstream; it supports calcium and phosphate metabolism, indirectly influencing bone health [35]. In a previous study with postmenopausal women, the T allele of rs4588 was associated with lower VDBP and bone mineral density [36]. Interestingly, we found the same allele involved with a decrease in FD in the mandibular condyle.

SEC23A is a critical regulator of collagen secretion and skeletal development, playing a major role in bone matrix formation and osteoblast function by facilitating the proper trafficking of collagen‐rich vesicles from the endoplasmic reticulum to the Golgi apparatus. This process is essential for the synthesis and extracellular deposition of collagen, which serves as the primary structural component of the bone matrix. Additionally, SEC23A is crucial for maintaining osteoblast differentiation and function, ensuring the continuous production and organization of collagen fibers necessary for bone strength, mineralization, and overall skeletal integrity. Mutations in the SEC23A gene have been linked to skeletal abnormalities and disorders associated with impaired collagen transport [37]. In our study, rs8018720 in SEC23A was associated with FD. This SNP was previously involved in vitamin D circulating levels in a large genome‐wide association study [16].

PTH is well known for its critical role in bone metabolism, regulating calcium homeostasis, bone remodeling, and bone mineralization by influencing osteoclast and osteoblast activity. PTH plays a key role in maintaining serum calcium levels by stimulating calcium resorption from bones, enhancing intestinal calcium absorption via activation of vitamin D, and reducing renal calcium excretion. Additionally, PTH contributes to skeletal integrity by modulating bone turnover, ensuring a balance between bone formation and bone resorption to adapt to physiological demands. Some genetic studies support that SNPs in the PTH encoding gene can significantly influence bone metabolism by altering PTH secretion, receptor interactions, and downstream signaling pathways [17–19].

Fractal analysis of panoramic radiographs provides valuable insights into bone structural complexity. However, its limitations include image quality issues and geometric distortion due to the inherent two‐dimensional nature of radiographic images. Additionally, challenges in defining and standardizing regions of interest must be considered, as they can affect accuracy. These factors may limit the reliability of fractal analysis in panoramic radiographs, particularly when compared to more advanced imaging techniques like three‐dimensional computed tomography. Another important limitation of our study is that the type of skeletal malocclusion was not taken into consideration. The condyle adapts to functional stresses from mastication and occlusion. Skeletal malocclusion can lead to adaptive, structural, and functional changes in the mandibular condyle due to altered biomechanical loading, joint remodeling, and muscle function.

One important consideration should be highlighted here: in our study, only children were included. The mandibular condyle undergoes significant structural and functional changes from childhood to adulthood, reflecting its role in growth, remodeling, and adaptation to mechanical forces. The results observed in our study can differ in different age groups or ethnic groups. Our results may not be generalizable to other populations due to sample‐specific genetic, environmental, and demographic factors. Therefore, future studies across diverse populations are necessary, as evaluations in different settings are crucial for enhancing the generalizability of research findings.

## 5. Conclusion

The SNPs rs4588 and rs8018720 in vitamin‐related encoding genes (VDBP and SEC23A) and the SNP rs694 in the PTH encoding gene are associated with mandibular condylar trabecular bone structure in children.

## Conflicts of Interest

The authors declare no conflicts of interest.

## Funding

The study is supported by Coordenação de Aperfeiçoamento de Nível Superior (CAPES) Finance Code 001, Brazil, and the Alexander von Humboldt Foundation, Germany. Open access funding is enabled and organized by Projekt DEAL.

## Data Availability

The datasets used and/or analyzed during the current study are available from the corresponding author on reasonable request.
